# LATENCY/PATHWAYS, FUNCTIONALISM/MORPHOGENESIS: CONTRASTING PARADIGMS OF LIFE-COURSE INEQUALITY AND CHANGE

**DOI:** 10.1093/geroni/igz038.1560

**Published:** 2019-11-08

**Authors:** Dale Dannefer

**Affiliations:** Department of Sociology, Case Western Reserve University, Cleveland, Ohio, United States

## Abstract

Attention to dis/advantage during childhood has become a major interest of life-course studies. It has been a force in advancing attention to inequality over the undifferentiated “normal aging” versions of life-course and gerontological research, making clear the irreducible importance of the presence/ absence of key resources in accounting for life-course outcomes, from early onward. Explanatory strategies set forth within this work often contrast “latency/early origins” models (with explanation anchored in the early years) with “pathways” models (which examine the independent effects of adult life-course circumstances). This paper argues that these two types of models actually are aligned with distinct conceptual paradigms that imply fundamentally different understandings of aging in society (“functionalist/organismic” and “systemic/morphogenetic”). The differential implications of these two models for the relation of cumulative dis/advantage and social change is explored.

